# The Cenomanian/Turonian boundary in light of new developments in terrestrial palynology

**DOI:** 10.1038/s41598-023-30072-6

**Published:** 2023-02-22

**Authors:** Francesca Galasso, Ulrich Heimhofer, Elke Schneebeli-Hermann

**Affiliations:** 1 Paleontological Institute and Museum, University of Zurich, Karl-Schmid-Strasse 4, 8006 Zurich, Switzerland; 2 Institute of Geology, Leibniz University Hannover, Callinstrasse 30, 30167 Hannover, Germany

**Keywords:** Ecology, Environmental sciences

## Abstract

The Cenomanian/Turonian boundary interval is associated with an oceanic anoxic event (OAE 2,  94.0 Ma) during one of the warmest episodes in the Mesozoic. To date, plant responses to these climatic conditions are known only from the northern mid-latitudinal succession in Cassis, France. There, conifer-dominated and angiosperm-dominated vegetation types alternate. However, whether the exceptional environmental conditions had an impact on plant reproduction is unknown to date. We applied a new environmental proxy based on spore and pollen teratology on palynological samples from the Cassis succession, to explore if this phenomenon also occurs across the OAE 2. The observed frequencies of<1% malformed spores and pollen grains suggest that plant reproduction was not affected during the Cenomanian/Turonian boundary interval. While the effects of continental Large Igneous Province(s) on plant reproduction have shown to produce abnormal spore or pollen morphologies as evidence for severe environmental pollution, by contrast the effects of oceanic LIP(s) seems to be inconsequential.

## Introduction

Teratology is the science of malformation that occurs during the development of an organism. Spores and pollen are also known to show teratomorph morphologies, sometimes in frequencies exceeding the background values of plants grown under present-day natural conditions^1^.

In paleopalynology, teratomorphic spores and pollen have been successfully employed as a proxy for increased environmental disturbance. The occurrence of increased malformation in sporomorphs represents one of the more exotic proofs of stress response in biotas during the Devonian/Carboniferous boundary (DCB,^2^), the Permian/Triassic boundary (PTB,^3–5^), the Smithian/Spathian boundary (SSB,^6^), the Triassic/Jurassic boundary (TJB,^7–10^) and the Toarcian Oceanic Anoxic Event (T-OAE,^11,12^). Although much progress has been made in studying the vegetation response to environmental perturbations throughout the Mesozoic, the Cretaceous, particularly the Cenomanian/Turonian boundary, is still poorly studied and understood.

The early Late Cretaceous is one of those intervals in the Phanerozoic, which are characterised by “greenhouse” climate conditions^13^. During this period, ecosystems have experienced severe paleoenvironmental stress related to the warmest climate of the previous 150 Ma^14^. Especially the Cenomanian/Turonian transition (about 93.9 Ma,^15^) is characterised by profound paleoenvironmental changes on a global scale. The boundary interval is marked by an extinction event and turnover in planktonic foraminifera and radiolarians^16–21^, by an increase in sea level^22–26^, and by widespread dysoxic to anoxic conditions known as the Oceanic Anoxic Event 2 (OAE 2). The widespread anoxia-euxinia resulted in the depletion of isotopically light $^{12}$C in seawater due to increased organic matter burial, globally expressed by a positive carbonate excursion (CIE) in both marine and terrestrial organic and inorganic carbon (e.g.^27–31^). In addition to a major transgression, the time interval encompassing OAE 2 was dominated by oceanic anoxia and acidification and a biocalcification crisis where calcareous nannoplankton experienced anomalous paleoceanographic and paleoclimatic conditions that induced extinctions as well as originations (e.g.^14,20,32,33^). However, recently the decline of calcareous microfossils has been questioned by the observation of “ghost” nannofossils^34^. Along with the widespread anoxia, the onset of OAE 2 was accompanied by a rise in sea surface temperature (SST), known as the “Cretaceous thermal maximum”, which most likely occurred as abrupt warming associated with an increase in atmospheric *p*CO$_{2}$ (e.g.^24,35–40^). These warm conditions and high *p*CO$_{2}$ during the OAE 2 were interrupted by a transient period of climate instability (with multiple short SST drops and rises in the range of $2.5-11\,^\circ \hbox {C}$), re-oxygenation of the oceans, and a shift in the vegetation community, named the Plenus Cold Event (PCE) (e.g.^29,35,37,38,40–44^). The environmental changes and biotic responses during the OAE 2 have been tentatively linked to the emplacement of widespread submarine basaltic plateaus of large igneous provinces (LIPs; the Caribbean and High Arctic LIPs)^45–56^. LIPs released huge volumes of CO$_{2}$ and introduced high concentrations of biolimiting metals into the ocean and atmosphere^46^ that possibly resulted in ocean fertilisation and/or poisoning by toxic metals^20,33^ affecting both terrestrial and marine communities.

The sedimentary succession at Cassis (southeast France) has been well-calibrated by ammonoid^57^, planktonic foraminifera, and nannofossil biostratigraphy^58^, and $\delta$ $^{13}$ $\text {C}_{org}$ chemostratigraphy^38^. The palynological data of the Cassis section provides the first complete assessment of plant responses during the OAE 2^38^. Palynological studies in this region have identified two alternate vegetation types. (1) During the Cretaceous thermal maximum, the vegetation was characterised by a dominance of conifers growing in mesic habitats with moderate availability of moisture^38^, although the high temperatures might have played a critical stress factor on the vegetation and plant development, even by partially slowing photosynthesis with temperature exceeding $35-40\,^\circ \hbox {C}$^13^. (2) During the PCE, cooler climate and less humid conditions favoured open, savanna-type vegetation dominated by angiosperms^38^. However, the environmental proxy based on spore and pollen teratology has not been applied so far.

According to Green et al.^59^, the major Phanerozoic extinction events may be triggered by comparable mechanisms related to continental flood basalt emplacement. In light of the repeated observations of malformed sporomorphs during times of major biotic crises, in this contribution we evaluate whether teratomorphies occur during the Cenomanian/Turonian boundary interval and which potential causes ascribed to this phenomenon are reflected in the geological record.

## Geological setting

**Figure 1:**
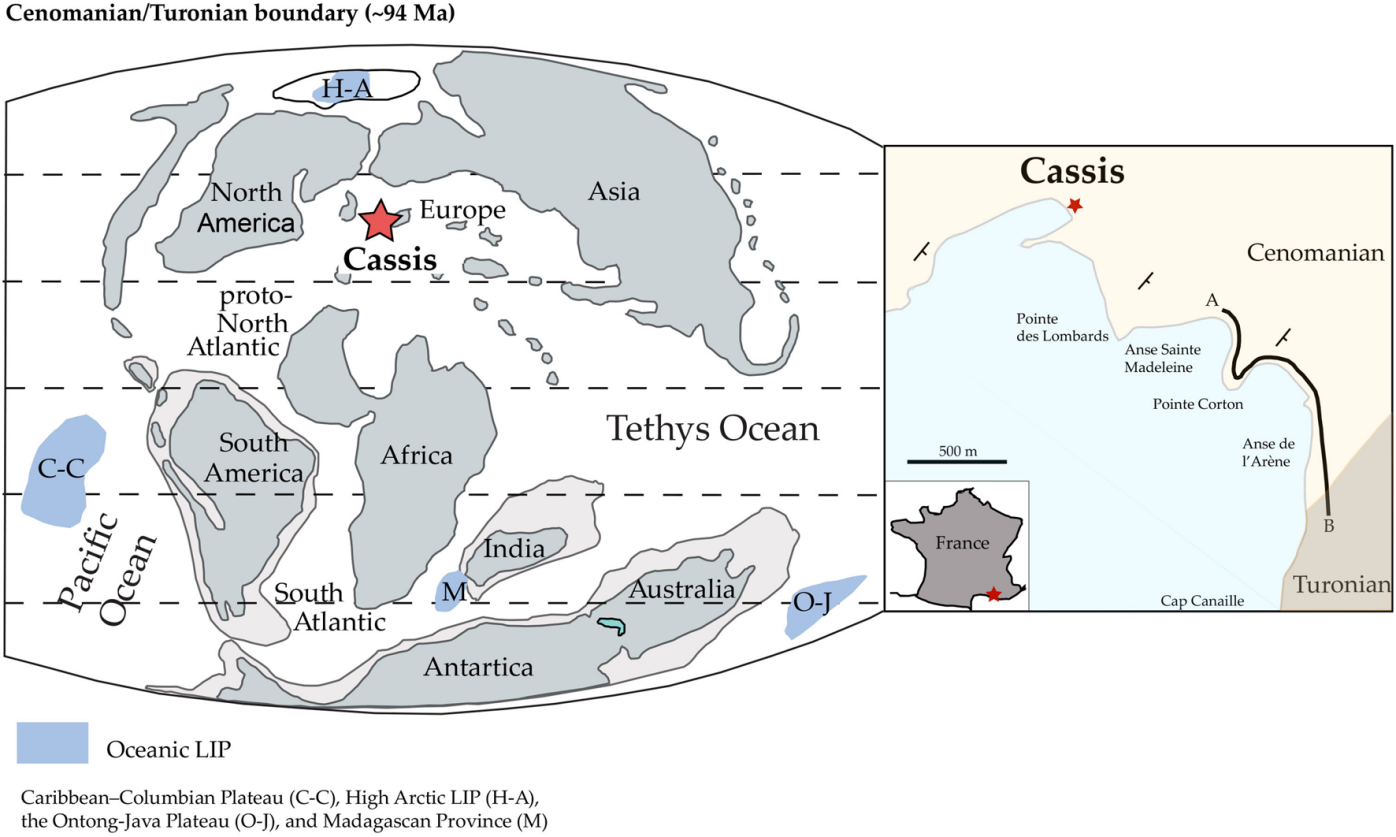
Simplified Late Cretaceous paleogeographical map, location map and the studied stratigraphic section (**A**, **B**). Figures modified after^57,60^, respectively.

The Cassis sedimentary succession is exposed along the French Mediterranean coastline, stretching from Pointe des Lombards in the town of Cassis towards the southeast to the foot cliff of Cap Canaille (Fig. 1).

**Figure 2:**
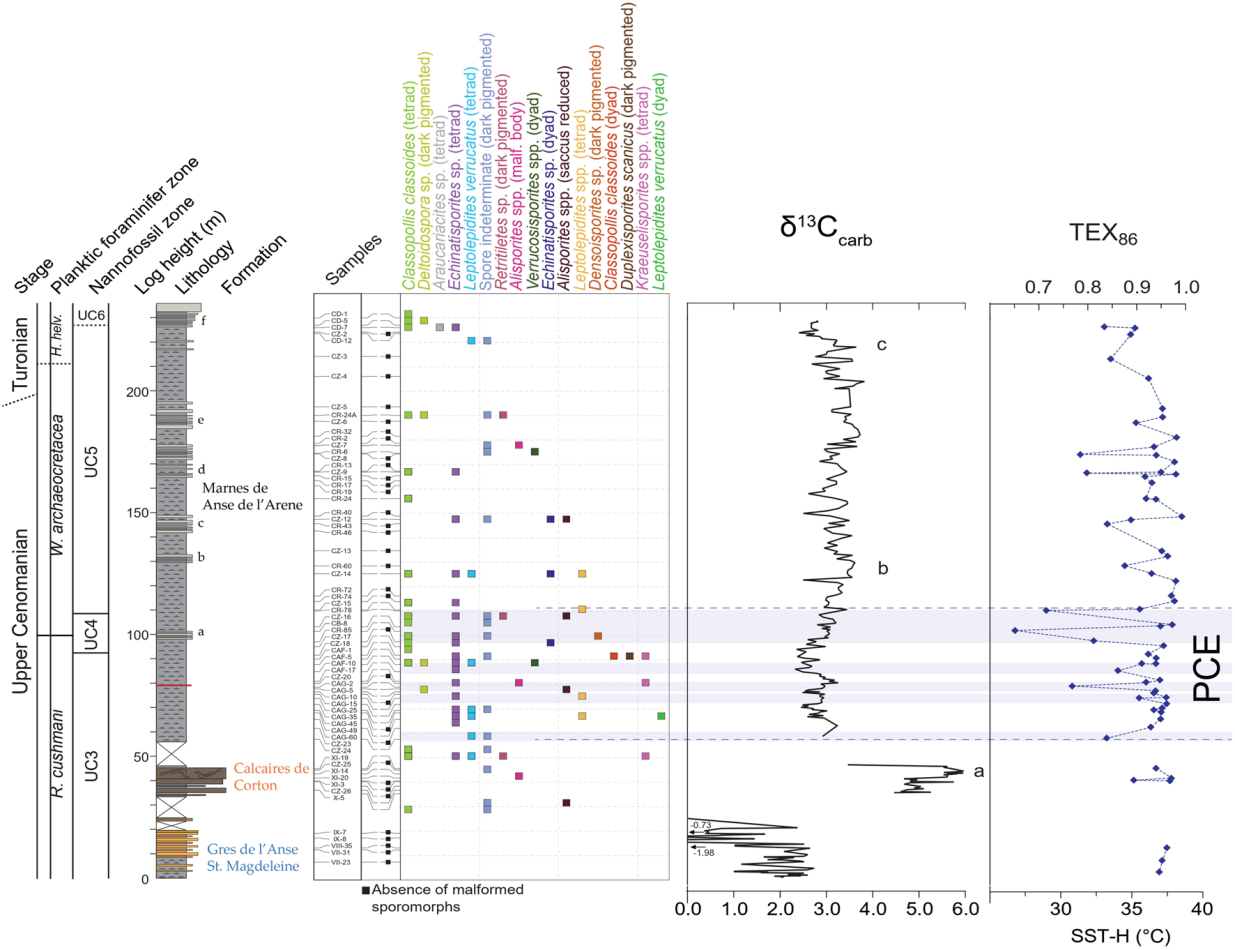
Stratigraphical distribution of malformed sporomorphs in the Cassis section. Presence/absence data of abnormal morphological variation in sporomorphs. Each cross represents a minimum of two occurrences of the respective sporomorph. Lithological log, biostratigraphy, $\delta$ $^{13}$ $\text {C}_{carb}$, $\delta$ $^{13}$ $\text {C}_{org}$, TEX_86_-derived sea-surface temperatures (SSTs) adapted from^38^. PCE = Plenus Cold Event. Blue horizontal bands represent individual SST cooling episodes during the PCE.

The section is  235 m thick and encompasses the Grès de l’Anse Sainte Magdeleine Fm. and the Calcaires du Corton Fm. (lower upper Cenomanian), and the Marls de l’Anse de Arène Fm. (upper Cenomanian to lower Turonian). The base of the studied succession is located 500 m southeast of the Cassis marina at Pointe de Lombards ($43^\circ$12′ 34.0″ N $5^\circ$32′ 23.0″ E) and is characterised by a monotonous lithology of clays and marls, interbedded with turbiditic sandstones at the top of the Grès de l’Anse Sainte Magdeleine Fm. (0–19.7 m) (Fig. 2), followed by a 10-meter-long gap in the outcrop. The overlaying Calcaires du Corton Fm. (33.4–46.1 m) is characterised by calcareous marls and limestones with intense slumping structures of the Calcaires du Corton Fm., subsequently interrupted by a second gap. The upper part of the section (55.8–236.0 m) is represented by continuous grey marls deposits and a series of nodular limestone layers (labelled a-f in Fig. 2) corresponding to the Marls de l’Anse de Arène Fm. For further in-depth information on the Cassis section’s sedimentology, chemo- and biostratigraphy, see^38^.

## Results

**Figure 3:**
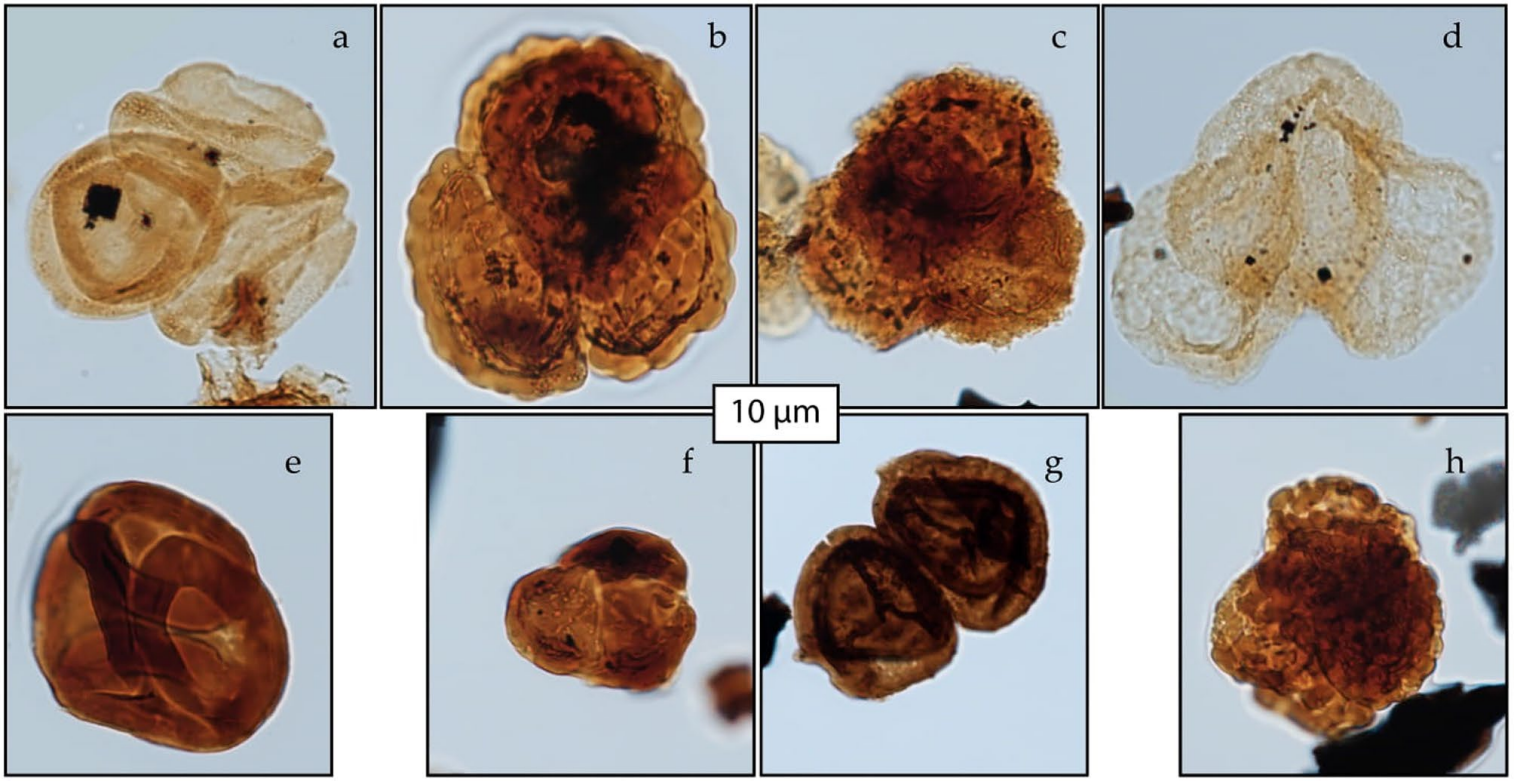
Selected spores and pollen from the OAE 2 of Cassis (southeast France). The sample number is followed by England Finder coordinates.**(a), (f)=** ***Classopollis classoides*** **(tetrad)**, sample CAF 10, V51/4; CB 8, O34/1; **(b), (h)=** ***Leptolepitides*** **sp. (tetrad)**, sample CAG 35, O30; XI-M-19, K36/1; **(c)=** ***Kraeuselisporites*** **sp. (tetrad)** sample CAF 5, Y35; **(d)=** ***Alisporites*** **sp. (expanded central body and folded left saccus)**, sample CAG 2, X50; **(e)=** ***Classopollis classoides*** **(welded grains tetrad)**, sample XI-M-19, O41/3; **(g)=** ***Densoisporites*** **sp. (dyad)**, sample CZ 17, U43.

The preservation of palynomorphs in the Cassis section varies markedly, ranging from moderate to good. The rich and diverse spore-pollen assemblage of the Cassis section is dominated by inaperturate and bisaccate gymnosperm pollen produced by Araucariaceae and Cupressaceae-type plants (*Araucariacites* spp., *Inaperturopollenites* spp.), Cheirolepidiaceae (*Classopollis* spp.) and by angiosperm pollen of the Normapolles-type-group, including *Atlantopollis* and *Complexiopollis*. On the other hand pteridophytes produced by a diverse assemblage of ground ferns and fern allies were present in a negligible amount. The first shift in the floral association occurred with the inception of the PCE where a dominance of Normapolles-type pollen (e.g. *Atlantopollis microreticulatus*) coincides with the decline of conifer pollen contents (e.g. *Inaperturopollenites* spp., see Fig. 3 (AZ III) in^38^). With stratigraphic height, the spore-pollen assemblage shows a major change in frequency distribution patterns including a pronounced increase in *Inaperturopollenites* spp. followed by a decline in certain species of *Atlantopollis* (pronunced in *Atlantopollis microreticulatus*), see Fig. 3 (AZ VI) in^38^.).

After a thorough screening of the palynological slides of the Cassis section, almost no malformed sporomorphs were found, except for the seldom occurrences of spore/pollen tetrads and dyads (Fig. 3). Spore dyads, including *Densoisporites* spp. and *Leptolepidites verrucatus*, were found in a single arrangement—two grains close/attached one to the other. Furthermore, spore tetrads of the taxa *Echinatisporis echinoides*, *Leptolepidites* spp., *Kraeuselisporites* spp., and *Retitriletes* spp., as well as pollen tetrads such as *Araucariacites* sp., and *Classopollis classoides* were found as welded grains or as grains close/attached to one another. Despite changes in plant community, such as the radiation of angiosperms and the proliferation of cold and less humid savanna-type vegetation^38^ coinciding with the PCE, we acknowledge the presence of teratological lycophyte spore tetrads, i.e. *Echinatisporis* spp. and *Leptolepidites verrucatus*. Furthermore, *Leptolepidites* spp. was also recorded in a single occurrence as a spore dyad in sample CAG-35. Lycophytes, as well as ferns, are best represented in environments with high humidity and moderate temperatures in tropical and subtropical forests^61^. However, many species are also morpho-ecophysiologically adapted to low water availability and extreme temperatures^62^. The variety of habitats in which ferns and lycophytes can occur reflect their diverse strategies in response to environmental pressures^63^.

*Classopollis classoides* in the Cassis section had somewhat comparable sizes to dispersed *Classopollis*, which ruled out polyploidy^11,12,64^. It is known to frequently occur in tetrads^65^ in low-energy shelf environments or oceanic sediments that were deposited far from land^66,67^. As a result, the presence of *Classopollis* tetrads is not necessarily a sign of environmental disturbance. Although the genus is commonly associated with semiarid to arid climates (e.g.^68^), it has also been described from coastal environments or areas subject to repeated volcanic ash fall, indicating its ability to adapt to dry, saline, or even disturbed habitats (e.g.^69,70^). Besides, the presence of *Classopollis classoides* tetrads during the PCE contrast with the disappearance or sporadic occurrence of the taxon described from the nearby Pont d’Issole section (Unit Th2)^71^.

Along with changes in size and form, we also noticed sporomorph color variations in the Cassis section. While *Leptolepidites* spp. and other spores with thick and ornamented sporomorph wall were ignored, spores with medium and smooth exine, such *Deltoidospora* spp., had occasionally a darker color. This peculiar characteristic, though, could be the result of a taphonomic artefact rather than environmental stress; therefore, further studies are needed to validate this external trait. The few occurrences of spore dyads and tetrads were less than 1% on average and, therefore, below the baseline frequency of 3-5% above which environmental influence on spore and pollen morphology can be assumed^1,8,12,72^. They reflect the natural variation of spore and pollen morphology (e.g.^73,74^). However, the stratigraphic frequency distribution of sporomorph malformations and tetrads has revealed a buildup of these malformations coinciding with the PCE (Fig. 2). As opposed to sporomorphs, a complete lack of malformation was found in another palynomorph group- the angiosperms. While polyploidy is an unusual phenomenon among extant gymnosperm, it is common in flowering plants^64,75^. Angiosperm might have been unaffected by the harsh environmental conditions experienced at the OAE 2 by applying a successful strategy as hybridization or polyploidizations (whole-genome duplications).

## Discussion

The Earth’s climate has changed dynamically over geological time, oscillating between two primary states: the “Icehouse” state and the “Greenhouse” state. The climate in each of the states varied significantly, and additional driving forces modified climatic conditions, either leading to cooler or warmer climates. Large igneous provinces (LIPs) are enormous crustal emplacements (millions of km$^{3}$) of predominantly mafic extrusive and intrusive rocks originating via processes other than “normal” seafloor spreading, e.g. continental flood basalts (or traps), volcanic passive margins, oceanic plateaus, and seamount groups^76^. The emplacement of LIPs can be related to hyperthermal events in Earth history. For example, the Siberian Traps are associated with the Permian/Triassic boundary, the Central Atlantic Magmatic Province with the End Triassic, the Karoo-Ferrar with the Toarcian OAE and the Caribbean and the High Arctic with the Cenomanian/Turonian boundary, among others. Comparing various LIPs and associated environmental and biotic responses, e.g. the Permian/Triassic boundary (PTB,  252 Ma), the Smithian/Spathian boundary (SSB  249.2 Ma), the Triassic/Jurassic boundary (TJB,   201.51 Ma), the early Toarcian oceanic anoxic event (T-OAE,  183 Ma), and the Cenomanian oceanic anoxic event (OAE 2,  94 Ma) the severity of their effect on climate and biota differs. The PTB, SSB, TJB, and T-OAE are associated with continental LIPs. Proxy evidence for volcanism in stratigraphic strata points to a strong temporal correlation between LIP volcanism and climate warming, reflecting the beginning of environmental change^77–83^. Continental LIPs eruptions caused significant changes in temperature, habitat, and biota in both marine and continental settings. The dramatic $\delta$ $^{13}$ $\text {C}$ anomalies were attributed to the catastrophic release of highly volatile methane hydrates originating from intruded volatile-rich sediments such as organic-rich shales, coals, and evaporates by LIP magmas^84–86^, leading to a runaway greenhouse. This is, in turn, assumed to have been triggered by the massive release of volcanic CO$_{2}$. Depending on the individual components of volcanic gas release, the effects on the atmosphere and continental environments involved global warming, an increase in wildfires and droughts, intensified continental weathering and terrigenous input, acid rain, ozone layer destruction, metals poisoning, and cooling, which caused profound changes on land (^82,87^).These changes include for instance, a shift in plant-community dominance structure from pollen-dominated to a spore-dominated ecosystems (i.e. spore “spike”) (e.g.^88–94^), and the occurrence of malformed pollen and spores beyond background values (e.g.^2,5,7–12^). In both cases, the harmful effects of LIPs, such as halocarbons, SO$_{2}$, and phytotoxicity, were ascribed as the potential causes^7,8,95^. By contrast, the OAE 2 resulted from submarine LIP emplacement on oceanic crust.The OAE 2 was associated with two LIPs, including the Caribbean-Columbian Plateau and the High Arctic LIP^45–56^. The effects of submarine LIPs eruption on the environment included widespread anoxia, the large-scale burial of organic matter, toxic metals, global warming or cooling and the partial demise of carbonate platforms. Given that the volcanism occurred predominantly below the sea surface, the effect of released gases was buffered by seawater^82^. Therefore, the impact on the deep-sea biota was much more severe than on shallow marine and terrestrial biota. Compared to other major biotic crises in the Earth’s history, the OAE 2 has shown an extremely low abundance of malformed spores and pollen. This suggested that likely variations between different types of LIPs in terms of emplacement and eruptive style (e.g. submarine vs subaerial volcanism and additional thermogenic emissions) caused different responses of global vegetation. In summary, the Cenomanian/Turonian boundary succession in Cassis revealed only a few malformed spores and pollen grains. The extremely low abundance of teratomorphs found across the OAE 2 indicates only minor stress-related effects on land plant reproduction. Perhaps the mode of LIP emplacement and the associated volatiles coinciding with the Cenomanian/Turonian boundary interval were insufficient or not active to disrupt the terrestrial community or to cause malformation in land plant reproductive structures.

## Materials and methods

For the initial palynological study of the Cassis section, a total of 67 rock samples were prepared by the Geological Survey of North Rhine-Westphalia in Krefeld, Germany. To remove carbonate and silica, cleaned, crushed, and weighed samples (20 to 50 g) were subjected to 30% HCl and 38% HF treatments, respectively, according to the standard palynological preparation^65^. Residues were sieved over an 11-$\upmu \,\hbox {m}$ mesh and mounted on microscope slides.

After a rigorous palynological screening of the Cassis section, this research aimed to evaluate the occurrence and the abundance of malformed features in spores and pollen grains across the OAE 2. Following the method established by Galasso et al.^6^, we attempted to quantitative and qualitative describes morphological traits in sporomorphs and discriminate between alterations caused by preservation and/or those caused by malformation across the Cenomanian/Turonian boundary.

## Data Availability

All data generated and/or analysed in this study are included in this published article.
